# Conscience and Consciousness: a definition

**Published:** 2014-03-25

**Authors:** G Vithoulkas, DF Muresanu

**Affiliations:** * International Academy of Classical Homeopathy, Alonissos, Greece; ** “Iuliu Hatieganu" University of Medicine and Pharmacy, Department of Neurosciences, Cluj-Napoca, Romania

**Keywords:** consciousness, neuronal correlate of consciousness, neuroplasticity, conscience, free will

## Abstract

While consciousness has been examined extensively in its different aspects, like in philosophy, psychiatry, neurophysiology, neuroplasticity, etc., conscience though it is an equal important aspect of the human existence, which remains an unknown to a great degree as an almost transcendental aspect of the human mind. It has not been examined as thoroughly as consciousness and largely remains a “terra incognita" for its neurophysiology, brain topography, etc. Conscience and consciousness are part of a system of information that governs our experience and decision making process. The intent of this paper is to define these terms, to discuss about consciousness from both neurological and quantum physics point of view, the relationship between the dynamics of consciousness and neuroplasticity and to highlight the relationship between conscience, stress and health.

## Consciousness

The meanings of the two terms “conscience" and “consciousness" are often confused and are misunderstood by many people.

 This article is an effort to clarify these meanings and to show the role of a “clear conscience" or a “troubled conscience" in health and disease.

 “Consciousness" is the function of the human mind that receives and processes information, crystallizes it and then stores it or rejects it with the help of the following:

1. The five senses

 2. The reasoning ability of the mind

 3. Imagination and emotion

 4. Memory

 The five senses enable the mind to receive information, then imagination and emotion process it, reason judges it, and memory stores or rejects it.

 The exact parts of the human brain [**1**] where those functions take place are supposedly defined by neurophysiology [**2**]. An important observation is that the more information one is able to gather and process, the more “aware" and the more “conscious" one becomes regarding one’s internal and external world [**2**]. Awareness and wakefulness represent the two main components of consciousness. Awareness is defined by the content of consciousness, and arousal is defined by the level of consciousness. Awareness contains self-awareness, which perceives the internal world of thoughts, reflection, imagination, emotions, and daydreaming, as well as external awareness, which perceives the outside world with the help of the five senses. From a neurological point of view, consciousness comprises a spectrum of states that range from physiological states to states of impaired consciousness that are monitored by specific criteria included in the Glasgow Coma Scale but also comprises modified states either by self-training (transcendental meditation) or by drug intake.

 Neuroanatomical studies revealed numerous structures implicated in the consciousness that were very well described by De Sousa’s remarkable review on the multidimensional concept of consciousness [**3**]. An essential structure that mediates the arousal is the ascending reticular activating system (ARAS) that comprises neurotransmitter-specific fibres from the reticular nuclei of the brainstem that are connected to the cortex via thalamic and extra-thalamic pathways and projecting to the hypothalamus and basal forebrain [4,5]. Following the ARAS, other important structures in consciousness are the amygdala, which modulates memory, attention, emotion and higher cognitive functions, as well as the cerebellum, which modulates executive function, cognition and emotion [**6**]. Both the prefrontal cortex and precuneus seem to be correlated with self-perception and metacognition [7,8]. Additionally, the precuneus and prefrontal cortex along with the temporoparietal junction and anterior cingulate gyrus represent areas implicated in the “default mode" of brain function during the conscious resting state [**9**]. Frontoparietal connectivity and the thalamus are considered the most important neural correlates of consciousness. Frontoparietal connectivity is implicated in maintaining awareness, in attention and in behavioural selection of incoming and stored information [**10**]. The thalamus is the final relay station for perceptual data before reaching the cortex. It also plays a key role by modulating cortical activity [**11**]: the thalamus and cortex are connected in a reciprocal manner and this connection seems responsible for higher cognitive processes. Moreover, the thalamic reticular nucleus (TRN) appears to control thalamocortical synchronization [**12**].

 A very different theory from that of a neural correlate of consciousness, which assumes that consciousness is a single unified entity, is the theory of multiple consciousnesses with three hierarchical levels: micro-consciousness, macro-consciousness, and the unified consciousness [**13**].

 One of the multiple theories of micro-consciousness considers that the functional unit of consciousness consists of a triangular neuronal configuration, the assembly of which is unconstrained by conventional anatomical boundaries. These assemblies vary in size from one moment to the next, with every moment being correlated with different degrees of consciousness. The complexity and dimension of these assemblies depend on the synchronicity of their synapses (known as Malsburg synapses), the strength of the trigger that initiates their transient synchrony, and on the availability of neurotransmitters [3,14]. 

 Beyond the neurological descriptions of consciousness that consider that consciousness is generated at the neuronal level is the quantum physics approach, governed by classical physics, which confers a more dynamical vision but which also gives rise to several controversies [**15**]. According to the quantum physics view, consciousness depends on self-observation. It is continuously self-creating by unconscious processes that are constantly coming into existence through self-awareness, such as the act of observing an electron, which concretizes that electron by collapsing the wave function [**16**]. This image of consciousness allows for the coexistence of “multiple, half-formed ideas, all flitting below the threshold of awareness at the same time" waiting for the self-observing process to end this superposition and to concretize a singular idea [**17**]. Such a dynamical construct implies a continuous change in the brain’s organization. Neuroplasticity and consciousness are bi-directionally connected: with consciousness, on the one hand, being the result of the growing complexity of the connection of some activity and, on the other hand, reorganizing brain connections through learning activities [**18**]. The conscious brain is in a perpetual state of learning. It learns how to describe and re-describe its own activity to itself, developing complex systems of metarepresentations [**19**]. Also, the dynamic impact of consciousness upon brain connectivity continues beyond wakefulness, with dreaming also having an important impact upon neuronal networks [3,20]. 

 Another important aspect of neuroplasticity in consciousness is represented by the modified state of consciousness during mindfulness process. From the neuroscience point of view, focusing attention practice produces measurable changes in spontaneous brain activity by increasing gamma frequencies [21,22]. These electromagnetic changes are substantiated by imaging studies that demonstrated both dynamic white matter changes like increased myelination and connectivity [**23**] and increased cortical thickness [**24**]. 

## Conscience

We must remember that the mechanisms of “consciousness" are complex and intricate, whereas the workings of “conscience" are much simpler. The concept of “conscience", as commonly used in its moral sense, is the inherent ability of every healthy human being to perceive what is right and what is wrong and, on the strength of this perception, to control, monitor, evaluate and execute their actions [**25**]. Such values as right or wrong, good or evil, just or unjust, and fair or unfair have existed throughout human history but are also shaped by an individual’s cultural, political and economic environment 3. The closer our inner state of conscience identifies with the higher perception of these concepts, such as good, right, just, and fair, the higher our degree of “conscience", and less physical stress is experienced if we feel that we act according to these concepts 4. It can be said that “conscience" 5 is the degree of integrity and honesty of each human being because it monitors and determines the quality of one’s actions. One who acts with a “clear conscience" has the advantage of feeling inner peace, which is a feeling that mitigates the adverse physiological effects experienced in times of stress. Conscience is the “highest authority" and evaluates information to determine the quality of an action: good or evil, fair or unfair and so on. Consequently, conscience ranks higher than consciousness and, in addition, has the ability and the authority to decide how information will be used, either for good or for evil. However, conscience is usually influenced by and modified in its decisions by the natural instincts of humans for “survival" and “perpetuation". In other words, conscience determines our final decisions for action after evaluating, in a split second, all of the above parameters [**7**].

## The “systemic function" of the brain

This whole process (information-consciousness-awareness-conscience) should be understood in its entirety as a complex, continuous and integrated set of functions in all healthy human beings. If any part of these functions is defective or ceases to exist, the whole system will suffer or may even collapse. This demonstrates the wholeness, coherence and continuity of the human brain’s structure, and it means that, even though we theoretically can distinguish between functions for the purposes of research and understanding, these functions in fact operate as a systemic whole with an absolute interdependence between the above-mentioned parts. 

## Free will

We can decide to act in accordance with or against our conscience at any given moment. 

 Indeed, those are our only options. It is only within this framework that “freedom of choice" can exist. This means that decisions and actions that are in accordance with the dictums of the individual's “conscience" can lead to an evolution and refinement of conscience, as a result of an inner peace of mind. Such is the effort of all truly spiritual people. On the contrary, if one acts against one's own conscience, it can lead to an “involution" and a feeling of having a troubled conscience. In such a case, the overall “director and judge" becomes less distinct or even quiescent; its voice cannot be “heard," and it allows the lower instincts to gain the upper hand and to act accordingly. In this condition, a process begins that creates an inner “irritation", or inner “itch", that does not allow a moment of peace. Eventually, anxieties and phobias manifest, and they are the prodromal symptoms of a harassed health state. This happens in our contemporary societies, in which many initially healthy individuals who have become prominent figures, such as politicians, journalists, police officers and judges–those who have power over others in their hands but not enough moral strength–succumb to the widespread corruption of our times. Instead of using their power for the benefit of the people, they use it only for their own personal gain. This is not the case for all of them, of course, but those who want to go against such a trend find themselves eventually isolated and powerless. If conscience comes under pressure from the basic instincts and becomes dulled, then the human being will descend more and more into an animal-like state and will then be forced to exclusively serve his own lower instincts. In this compromised state, the information that an individual receives is assessed and utilized according to what is commonly called “self-interest", a term that has assumed the status of a “divine law" in our time. If any of the basic functions, like imagination, reason or memory, are diminished or lost due to some illness or injury, then the process of awareness suffers, and the whole system may eventually collapse. In such a case, conscience can no longer function. This happens in cases such as schizophrenia, Alzheimer’s disease, and severe brain injuries, for example. This leads us to the conclusion that the overall functional ability of the brain (information-consciousness–conscience) leads to decision and to actions. This ability’s characteristics are as follows: it has a hierarchical nature (its various functions are of higher or lower order). It has a unique character due to its infinite complexity, it is integrated (if a part collapses, the whole system may suffer or collapse), and it is continuously changing (new information is constantly absorbed, affecting and differentiating levels of conscience). The hierarchical capacity of the human brain to make final and meaningful decisions is responsible for whether a person decides to commit himself to the quest for God, as do the monks, adepts and mystics, or to the quest for Truth, as do the philosophers and scientists, or to deceiving others, as do the criminals. In this way, conscience formulates every level of experience, from the lowest to the highest, even to the transcendental and sublime.

 These transcendental, extramundane experiences of spiritual people can take place while the person is still in relatively good health and, at the same time, can understand and realize the ever-complex incoming information and thus make decisions and actions in split seconds. People who have managed to have a high level of conscience usually have a “higher purpose in life"; they have “visions that can inspire others" and aim always to help “others" or humanity as a whole. It is through such a process that a new quality of conscience eventually emerges to sacrifice self-interest for the common good. Experience has shown that those individuals who were raised in families with strong moral attitudes can very seldom bypass the dictates of their conscience. Conscience, being the noblest function of our existence, constitutes the thread that keeps us in contact with our universal nature or with the objective Truth or with God or whatever one wants to call it [**9**].

 Consequently, the definition of the “degree of conscience" anyone possesses can be determined as follows: it is the degree to which we “participate" in the objective Truth, namely the absolute Good or the absolutely “Right" or the absolutely “Just". Realistically speaking, humans cannot reach the absolute. They can only come closer to or go further from the absolute depending on the quality of their conscience. Unfortunately, this relative approaching of the Truth can change within the same person, sometimes in dramatic way. The degree of conscience, or how close the awareness of the person is to the Truth, depends, unfortunately, on two factors:

 a. The assessment of the information received

 b. The need of the individual to indulge their human instincts

 We are saying “unfortunately" because it is too easy for conscience to drop to a lower level if the choice of the person is for comfort and self-interest alone. On the contrary, it is too difficult to attain a higher level of conscience because the individual must have adopted already, through long personal struggles, the concept of “sacrifice" of personal interests and comforts to achieve an ever-ascending level of conscience.

 Conscience attains a higher level only when the “common good" is put above “self-interest" [**26**]. This happens in an almost deterministic way. Examples of high conscience are the adepts of all times, with their transcendental experiences, and all those who managed to tame their passions and pursue the search for Truth or all those who sacrificed their lives for the societies in which they lived. Examples of low conscience are those who managed to deceive, oppress and take advantage of not only a few people but of whole societies or nations for their own personal benefit. Such individuals are primarily the corrupted politicians whose actions may affect the whole nation. We, the common people, are somewhere between these two categories, and we fight tooth and nail to keep a somewhat balanced condition and not to shut down our conscience completely. It is a daily struggle, and we usually lose many battles; consequently, our health decreases until death completes the picture.

 Here, it should be noted that the action that brings the greatest catharsis and inner release is confession in a kind of public situation. The effects of psychological and psychotherapeutic treatments are based in this reality, whether it is admitted or not. The same fact has given power to all religions that have in their practices the act of confession. After an honest and deep confession, people have admitted that they felt rejuvenated and in better health. The decisions of people in positions of authority of all kinds depend on this individual state of conscience, whether their decisions will be destructive or constructive, affecting sometimes a whole nation or the whole of the planet. The dulling of their conscience is necessary for those in authority to find excuses for promoting their destructive measures as needful and constructive. Many aggressive wars, especially within the last 50 years, have been executed in the name of democratic ideals, while their victims included millions of people and they have caused innumerable others to suffer. This shows how unhealthy our leaders have become. An impressive book written by Prof. David Owen, “In sickness and in power. Illness in heads of Government in the last 100 years", depicts this idea exactly, as does the speech of Prof. J. Toole, “Neurological Health of Political Leaders" in the 2nd World Congress on Controversies in Neurology (Athens, 2008) [27,28]. Consequently, the more human beings tame their passions by distancing themselves from their basic instincts, the more their conscience evolves, reaching its highest level and giving the sense to the individual that they are living in a state of bliss. This evolution of conscience is an endless effort, one that goes on for as long as one lives; thus, in my opinion, conscience will never be defined as belonging to a certain part of the brain or as a chemically complex compound because the brain changes and evolves exactly because of those processes.

 We suggest that these concepts could formulate the “raw material" of a discussion that would examine whether conscience lies within the brain; whether it is only the result of a chemical compound or something different, lying beyond the brain structure, in a transcendental dimension; or whether both situations are necessary and true. 

 END

 1 Consciousness is also referred to, in the context of neurophysiology, as “subjective awareness" [**1**]

 2 The notion of consciousness or “subjective inner life" has also been addressed from a philosophical and religious point of view, with religious proposals being mainly metaphysical beliefs and philosophical proposals being theoretical speculative models [**2**].

 3 Surely, there are differences in the conscience of Eskimos, Japanese, Africans, Asians, Europeans, North Americans, and so on, such as differences regarding what is right and what is wrong with respect to particular life situations. However, all cultures know and agree on some basic concepts regarding morality.

 4 The formation of “conscience" through the ages is the highest spiritual characteristic of human beings. It was formulated through a complicated process of observation, experience in general, and suffering in particular. This particular stimulus for the development of disease should be a main theme in the teachings of medical institutions for learning and understanding diseases and their role in shaping conscience.

 5 In theology, a related notion to “conscience" is that of synderesis or synteresis, i.e., the habitual knowledge of the universal practical principles of moral action. While conscience is a dictate of practical reason deciding that any particular action is right or wrong, synderesis is a dictate of the same practical reason that has for its object the first general principles of moral action [**25**].

 6 In the field of moral conduct, there are various self-evident truths, which an average, normal person usually accepts, e.g., “do not to others what you would not wish to be done to yourself", “parents should be honored", etc.

 7 Examples of such cases are those who have a family that is starving and who steal, a criminal act, to save their own family from death. This is different from the one who steals public property to increase one’s own fortune. In the first case, though, the person may be jailed and can survive the ordeal without health consequences. The second person, however, will have to suppress their conscience from bothering them and will therefore have health consequences, as they fear that they may be discovered and have anxieties over what they have done.

 8 It is well known that, on a philosophical level, the concept of free will is very closely connected to the concept of moral responsibility.

 9 In addition, it is on a religious or a higher spiritual level that one can even speak of different types of conscience: a good conscience, an evil or defiled conscience, a weak conscience, a seared conscience.

 10 Robert K. Vischer of the University of St. Thomas School of Law in Minneapolis explores the legal notion of civil society as a moral marketplace where competing moral convictions and claims of conscience are allowed to operate and compete without invoking the trump of state power, thus allowing for a healthy and engaged public life [**26**].

 Conflict of interest.

 None declared.
